# Erratum

**DOI:** 10.4269/ajtmh.22-0547err

**Published:** 2023-07-05

After publication the authors identified inconsistencies regarding the units in their raw data regarding platelet and white blood cell counts. The paper has therefore been corrected. The results and conclusions are unchanged. A summary of corrections is below:

1. Platelet counts were updated in the RESULTS section (Page 71), FIGURE 2 (Page 72), and TABLE 1 (Page 72).
2. WBC counts were updated in TABLE 1 (Page 72).
3. The ROC for the platelets + WBC + retinopathy model was updated, from 0.89 to 0.87, in the RESULTS section (Page 72) and FIGURE 3 (Page 73).

The Supplementary Materials file has also been updated accordingly.

